# Research Progress on Moisture-Sorption Actuators Materials

**DOI:** 10.3390/nano14191544

**Published:** 2024-09-24

**Authors:** Dajie Zhang, Jia Ding, Yulin Zhou, Jie Ju

**Affiliations:** School of Nanoscience and Materials Engineering, Henan University, Zhengzhou 475004, Chinadj2944311925@163.com (J.D.); zyulin1204@163.com (Y.Z.)

**Keywords:** moisture-responsive materials, water-sorption, actuator

## Abstract

Actuators based on moisture-sorption-responsive materials can convert moisture energy into mechanical/electrical energy, making the development of moisture-sorption materials a promising pathway for harnessing green energy to address the ongoing global energy crisis. The deformability of these materials plays a crucial role in the overall energy conversion performance, where moisture sorption capacity determines the energy density. Efforts to boost the moisture absorption capacity and rate have led to the development of a variety of moisture-responsive materials in recent years. These materials interact with water molecules in different manners and have shown diverse application scenarios. Here, in this review, we summarize the recent progress on moisture-sorption-responsive materials and their applications. We begin by categorizing moisture-sorption materials—biomaterials, polymers, nanomaterials, and crystalline materials—according to their interaction modes with water. We then review the correlation between moisture-sorption and energy harvesting performance. Afterwards, we provide examples of the typical applications using these moisture-sorption materials. Finally, we explore future research directions aimed at developing next-generation high-performance moisture-sorption materials with higher water uptake, tunable water affinity, and faster water absorption.

## 1. Introduction

Moisture-sorption matters show high affinity to water molecules. Materials made of such matters can change their morphology or shape upon moisture environment. Based on these moisture-sorption materials, a lot of energy harvesting systems have been put forward, which provides a promising route to develop green energy and achieve a low-carbon economy [1,2,3,4,5,6]. The feasibility of moisture-sorption-based energy harvesting has been validated by a few documented moisture-sorption materials [7,8,9,10], although the energy conversion system suffers from the limitations of low energy conversion efficiency and power density. To promote energy harvesting efficiency, a variety of novel moisture adsorption materials have been developed. Correspondingly, many comprehensive reviews have emerged. For example, Qu et al. [11] detailed the working principles of a moisture-sorption energy harvesting system and summarized the progress in water sorbents. Wang et al. [12] systematically summarized the strategies and mechanisms of motile plant tissues in a humid environment. Yang et al. [13] introduced the mechanisms of humidity responsiveness of moisture-sorption materials and compared diverse strategies fabricating humidity-responsive liquid crystalline materials. Hu et al. [14] reviewed the research progress and design principles of carbon-based humidity absorption materials (e.g., carbon nanotube (CNT) and graphene oxide (GO)) with hydrophilic or hydrophobic functional groups. Zhang et al. [15] recapped newly designed moisture-sorption crystalline smart materials, such as molecular crystals and framework materials. They also gave an in-depth interpretation of the relationship between the structural characteristics and the responsive mechanisms of those materials.

Previous research findings have demonstrated the diverse types of traditional moisture-sorption materials. In recent years, new types and quantities of hygroscopic materials have emerged in abundance, such as liquid crystal polymers, graphene, the covalent organic framework (COF), the metal–organic framework (MOF), and so on [16,17,18]. Those materials demonstrate a macroscopic expansion or contraction through interactions with water molecules in different modes. A comprehensive review involving the different water absorption mechanisms and design principles of a moisture–energy conversion system is therefore highly desirable to aid in the real implementation of moisture-based green energy devices [19,20,21,22,23]. To this end, we summarize the recent progress in moisture-sorption energy conversion systems. We focus on the latest developments of moisture-sorption actuator materials, including biomaterials, polymers, nanomaterials, and crystalline materials. The design rationale, working principle, and critical issues in each type of these materials are discussed. Finally, the challenges and future research prospects for developing high-performance moisture-sorption actuators are explored. This review provides the guidelines for next-generation moisture-sorption actuators.

## 2. Mechanism of Moisture-Sorption Materials Responsiveness

**Figure 1:**
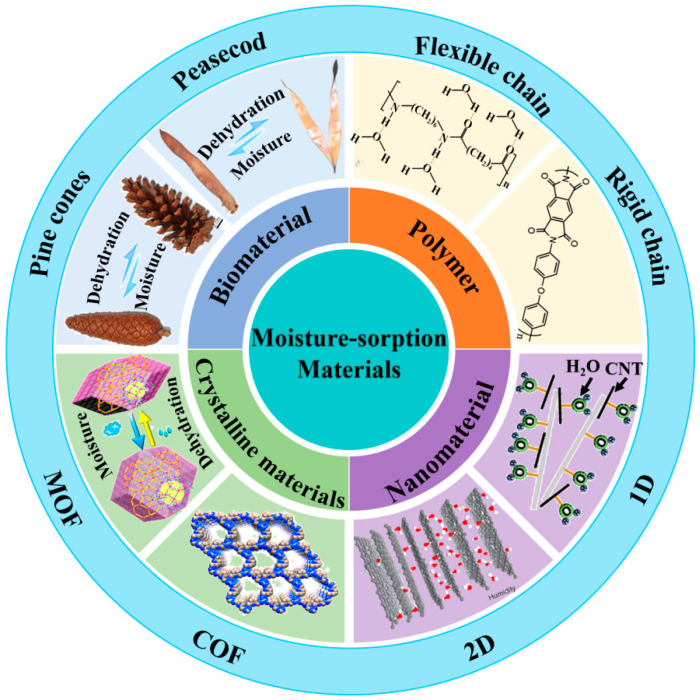
Various types of moisture-absorbing materials spontaneously undergo absorption and desorption processes, converting gaseous water from high-humidity environments into adsorbed water.

Figure 1 summarizes the types of moisture-absorbing materials [18,24,25,26,27,28,29,30,31,32,33,34]. The diversity of moisture-absorbing materials with different water affinities gives them high climate adaptability, making moisture-absorbing-based water phase change theoretically accessible anywhere and anytime.

### 2.1. Biomaterials

**Figure 2:**
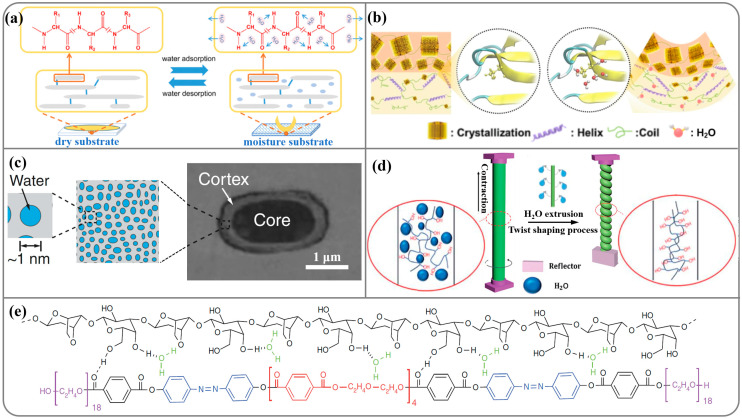
Mechanism of biomaterial humidity responsiveness. (**a**) The schematic of reversible water adsorption/desorption process of the soft biological film by extracting natural materials from pigskin. (**b**) Schematic drawing of the effect of water on gradient structure for moisture-driven silk fibroin films. (**c**) Spores exhibit a strong mechanical response to changing relative humidity by absorbing and releasing moisture. (**d**) Schematic diagram of the twisting process of the sodium alginate fiber. (**e**) Chemical structure of the agarose@poly(ethylene glycol)-conjugated azobenzene derivative (AG@PCAD) composite film and mechanism of water exchange.

Nature has always been a model of inspiration for technical developments [24,35,36,37,38,39,40]. Understanding the operating principles of natural actuators not only helps us to explore biological models with “smart” functions but also aids in the development of advanced artificial materials [41,42,43,44,45]. Natural biomaterials, such as collagen, silk fibroin, cellulose, chitosan, etc., are promising alternatives for fabricating flexible substrates owing to their permeability, biocompatibility, degradability, and implantation potential [46,47,48,49,50,51,52,53,54,55]. For instance, Li et al. [52] demonstrated the production of a biological film (BF) through the extraction of collagen from pigskin. Collagen is a central source of humidity-driven biomaterials. It denatures during hot water extraction, which leads to more random amino acid residues facilitating the formation of hydrogen bonds between the N–H group and the water molecules. In a high-humidity environment, it took 3 s for the BF to bend to a 120° angle and another 1 second to turn over, while at a lower humidity, it took as many as 10 s to bend to a 120° angle before the film stopped flipping. The BF, made of denatured collagen, is therefore more hydrophilic and moisture sensitive than the native pigskin. As shown in Figure 2a, the collagen chains respond to moisture by forming or breaking the hydrogen bonds in between to achieve water absorption or desorption. Apart from collagen, silk fibroin (SF), also derived from the natural material, shows an excellent moisture-responsive property. Molecularly, natural SF has a uniform semi-crystallinity and orientation. Zhang et al. [51] developed a strategy endowing an SF film with a gradient condensed structure from the very top surface to the interior. In a high-humidity environment, water molecules are absorbed from the bottom of the SF film, resulting in gradient stress across the gradient structure due to the recombination of the hydrogen bonds as shown in Figure 2b. The films were successfully origami-folded into a paper crane/boat without any damage and could realize the dynamic jump and moisture management functions.

The above two natural moisture-sorption materials demonstrate substantial advantages in high relative humidity (RH) conditions. At low and middle RH, materials capable of absorbing water have also been extensively studied. Goodnight et al. [56] reported bacterial spores exhibit strong hydration-driven actuation. As shown in Figure 2c, water confined to nanoscale cavities, conduits, and surfaces within hygroscopic materials can induce large pressures in response to changing relative humidity. An 8 μm thick polyimide tape coated with an ~3 μm thick spore layer changes its curvature in humid (uncurled) and dry (curled) conditions. When assembled in parallel, these tapes can lift weight against gravity in dry conditions. Wang et al. [57] twisted a gel-state natural alginate fiber through wet spinning, obtaining a fiber-based actuator that showed remarkable performance under water and moisture stimulations (Figure 2d). Owing to the excellent swelling and contraction properties in response to water, the twisted alginate fiber-based actuator underwent a rapid reversible rotational motion with a rotation speed of 13,000 rpm and a revolution of over 400 turns. Generally, the long-term stability of natural hygroscopic materials is limited by their relatively low mechanical performance. As shown in Figure 2e, Zhang et al. [21] prepared a composite film composed of strong hygroscopic capability of natural agarose and synthetic azobenzene (AG@PCAD), exhibiting high mechanical performance and rapid humidity responsiveness. In their design, the flexible diethylene glycol moieties provide elasticity, while the terephthalates functional groups are responsible for hydrogen bonding. The element can lift objects ~85 times heavier and can transport cargos ~20 times heavier than itself.

### 2.2. Polymers

Synthetic polymer materials exhibit exceptional molecular designability. The further incorporation of hydrophilic functional groups (e.g., hydroxyl (-OH), amino (-NH_2_), amide (-CONH-), carboxylic acid (-COOH), sulfuryl (-SO_2_-), sulfonic acid (-SO_3_H), and so on) into the polymer chains holds great potential for fabricating humidity-responsive novel materials with tunable water absorption and mechanical strength [20,58,59,60,61,62,63,64,65,66,67,68,69,70,71]. Comprehending the fundamental principles of structure design and synthesis of moisture-absorbing polymers is pivotal in the development of high-performance humidity-driven devices, which can significantly contribute to advancing intelligent energy conversion technologies.

**Figure 3:**
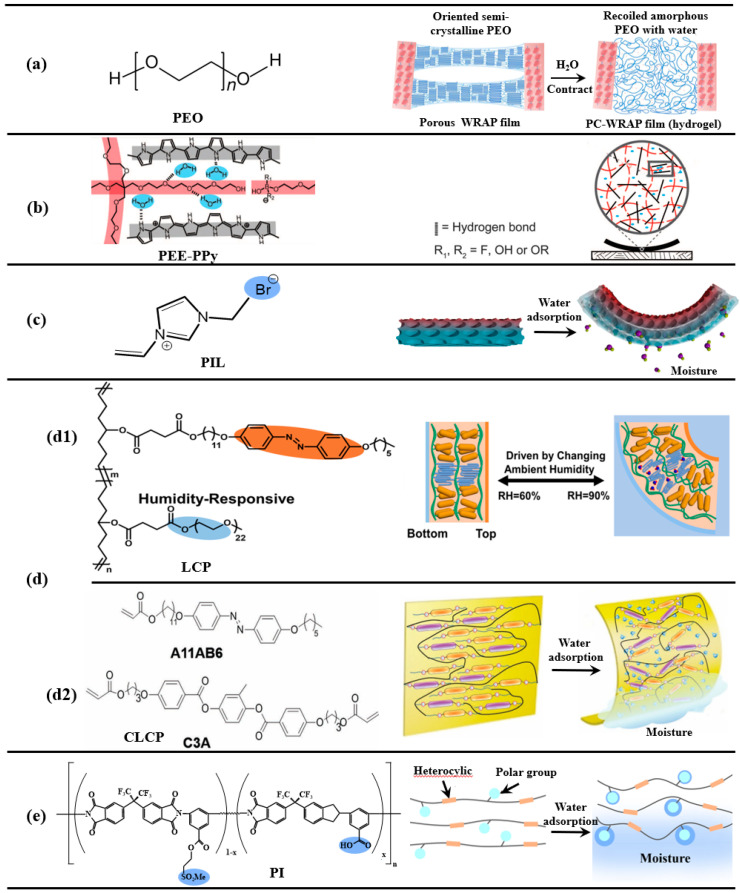
Mechanism of synthetic polymer humidity responsiveness. (**a**) Schematic diagram of the super-contraction mechanism of PEO semi-crystalline polymer for water absorption. (**b**) A PEE–PPy composite film (black) is composed of polypyrrole (PPy) polymer chains (gray lines) and a polyol-borate (PEE)-borate network (red lines). The structure changes (involving H bonds and borate ester bonds) in response to water (blue dots) sorption and desorption. (**c**) The fabricated inverse opal hydrophilic poly(ionic liquid) and sample bending upon absorbing water vapor. (**d**) Mechanism of synthetic liquid crystal polymer humidity responsiveness; Non mesogenic groups (**d1**) and mesogenic groups (**d2**) disrupted the ordered structure of the liquid crystal molecules. (**e**) The humidity response characteristics of polar groups in polyimide side chains.

Chen et al. [62] constructed a water-responsive shape-adaptive polymer (WRAP) film by orienting water-soluble semi-crystalline poly (ethylene oxide) (PEO) domains crosslinked by the poly(ethylene glycol) (PEG)-α-cyclodextrin (α-CD) inclusion complex crystalline domains (Figure 3a). Under uniaxial cold drawing, the PEO domains plastically deform to form aligned fibrillar bridges and a porous structure. Simultaneously, the PEO crystallites and chains orient and are temporarily fixed by the newly formed PEO crystallites. Water causes PEO chain recoil and super-contraction by destroying the PEO crystallites. After contraction, the PEO crosslinked by the inclusion complex becomes amorphous. Upon wetting, the WRAP film rapidly contracts by more than 50% of its original length within seconds. 

Langer and colleagues [20] made a dynamic polymer composite of rigid polypyrrole (PPy) imbedded with a flexible, interpenetrating polyol-borate (PEE) network that would be responsive to water sorption and desorption (Figure 3b). Intermolecular hydrogen bonding between the polyol-borate network and PPy also modulates the intermolecular packing of the polymer composite to alter its mechanical properties in response to water. The film actuator can generate contractile stress up to 27 megapascals, lift objects 380 times heavier than itself, and transport cargo 10 times heavier than itself. Wang et al. successfully developed a hydrophilic poly(ionic liquid) featuring an inverse opal porous structure [72,73], which remarkably enhanced the absorption of water vapor (Figure 3c). As a result, the film could flex at an astonishing angle of nearly 1440° within just four seconds. The orientation of liquid crystals (LCs) on the nanometer scale is easily manipulated by using alignment layers, and they are called liquid crystal polymers (LCPs) when fixed by polymerization. The deformation of LCPs depends on the ordered-to-isotropic alignment variation in the mesogenic units. Wei et al. [74] introduced hydrophilic non-mesogenic poly(ethylene glycol) (PEG) into azobenzene mesogenic units to obtain a humidity-responsive LCPs (Figure 3(d1)). However, introducing non-mesogenic groups into the LCPs disrupted the ordered structure of the liquid crystal molecules. Water vapor treatment facilitates the alignment of the LCs. Films prepared under high humidity (90% RH) showed uniform homeotropic alignment, while those prepared under lower humidity (60–80% RH) exhibited non-uniform alignment. The well-prepared films exhibit humidity-responsive bending, which is activated by the gradient humidity field or the changed ambient humidity. The humidity response behaviors are all attributed to the incorporation of hydrophilic moieties. The bending angles of the films increased with the increasing surrounding humidity, and the maximum deformation angle was 90° when the ambient humidity reached 95% RH. Yu et al. [75] demonstrated that a hydrophobic, porosity-free crosslinked liquid crystal polymer (CLCP) film without hydrogen-bonded LCs can undergo rapid macroscopic deformation in response to a humidity gradient. By means of hydration of the C=O and C-O-C groups in the LCs, the film swells and the alignment of the mesogenic units changes, causing the bending of the whole film in the direction perpendicular to that of the alignment (Figure 3(d2)). The relative actuation was observed to be dependent on the molecular configuration. The CLCP film with excellent humidity-responsive properties was designed to demonstrate a worm-like motion stimulated by humidity gradients; the moving speed of the CLCP “worm” was about 45 mm/min.

In addition, Tan and colleagues [76] successfully incorporated highly polar pendants (ester-sulfone and carboxylic acid) to a relatively stiff polyimide backbone. The polar groups such as the sulfonyl and carboxylic acid groups increase the polymer’s ability to absorb the moisture. The stiff polyimide films show the highest water uptake (~4.8%). Upon being laid flat on a piece of water-wet paper towel, two opposite parts of the film were able to curl up, followed by a quick roll-over and flattening action. Their combinations showed a remarkable vapor-gradient actuation by demonstrating numerous oscillatory cycles and locomotion on moist surfaces without performance degradation during storage (Figure 3e). Their work showed a simple, wholly covalent, and amorphous polymer in monolithic form, which can be hydromorphic and motile. The humidity-gradient responsivity they demonstrated would enhance the functional versatility of relatively stiff polymers. 

### 2.3. Nanomaterials

Nanomaterials have increasingly demonstrated their potential as highly suitable candidates for the development of soft intelligent actuators, owing to their exceptional electrical, thermal, optical, and mechanical characteristics [50,69,77,78,79,80,81,82,83,84]. For example, carbon nanotubes (CNTs), possessing a one-dimensional (1D) tubular structure, are widely acknowledged for their expansive specific surface area, exceptional electrical conductivity, remarkable mechanical strength properties, and an inverse axial thermal expansion coefficient. Graphene shows a two-dimensional (2D) carbon monoatomic layer structure. When subjected to treatments such as oxidation and irradiation, the various derivatives of graphene display diverse physical properties, including the photothermal effect and distinct variations in hydrophilicity and conductivity. Among those derivates, GO exhibits strong hydrophilicity and subsequently high humidity sensitivity as well as great dispersibility in water by a reduction process to remove the oxygen-containing functional groups and restore hydrophobicity thus losing dispersibility. In addition, MXene (Ti_3_C_2_T_x_) is a new 2D material with hydrophilicity, endowing it with versatility in the design of moisture-responsive actuators and sensors [9,36,43,64].

**Figure 4:**
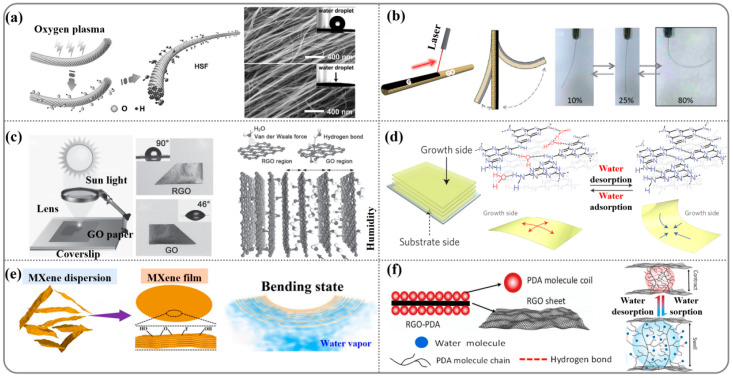
(**a**) Preparation of hydrophilic secondary fiber (HSFs) with hierarchically helical channels by oxygen plasma treatment. (**b**) The laser-assisted reduction in GO fibers and its deformation under different humidity levels. (**c**) Schematic illustration for the fabrication of GO/RGO bilayer papers using focused sunlight reduction, and mechanism of GO/RGO papers humidity responsiveness. (**d**) Schematic illustration of a possible mechanism for efficient conversion of water desorption/adsorption events into the bending and straightening motions of a carbon nitride polymer (CNP) film, respectively. (**e**) Schematic of the bilayer structure and the deformation of homogeneous MXene film actuator. (**f**) Schematic illustration of the fabrication process and mechanism of a uniform RGO–PDA thin film actuator.

The hydrophobic nature of carbon nanotubes is widely recognized. Peng et al. [85] successfully transformed these hydrophobic CNTs into hydrophilic ones through oxygen treatment, enabling the preparation of humidity-responsive actuators with rapid and reversible contractive actuation (Figure 4a). Upon coming in contact with a water droplet, a contractive stress of approximately 10.8 MPa was rapidly generated by the hydrophilic primary fiber (HPF) within 400 ms. It rapidly returned to the original state after removal of the water droplet. Qu et al. [86] fabricated graphene/graphene oxide (G/GO) (hydrophobic/hydrophilic) fibers, which are region asymmetric because of the positioned laser reduction in freshly spun GO fibers. Generally, the obvious change in surface wettability could be mainly attributed to the drastic removal of hydrophilic oxygen-containing groups, such as the hydroxyl, epoxy, and carboxyl groups. The GO adsorbs water molecules due to the formation of hydrogen bonds, whereas graphene adsorbs water molecules through the much weaker van der Waals forces. Therefore, the asymmetric G/GO fiber developed in this study should behave as a moisture-sensitive fiber actuator (Figure 4b). As expected, a rapid bending to the G side occurs once the G/GO fiber is exposed to moist air with a relative humidity (RH) of 80%, while the fiber recovers to the initial state when it is returned to the ambient condition. This process is fully reversible with an average motion rate of approximately 8°/s. Sun et al. [32] fabricated a graphene oxide/reduction graphene oxide (GO/RGO) bilayer paper using focused sunlight reduction (Figure 4c). In their scheme, the RGO side of the bilayer paper was hydrophobic, while the GO side was hydrophilic. As the humidity increased, the water absorption behavior of the GO nano-sheets caused the interlayer spacing to increase. Such an asymmetric swelling along the longitudinal direction of the GO/RGO bilayer paper accounts for its obvious moisture-responsive bending properties. Furthermore, with the increase in RH from 24% to 86%, the curvature of the GO/RGO ribbon increased gradually from 0° to 168°.

In addition, Aida et al. [87] reported a π-stacked carbon nitride polymer (CNP) with a highly anisotropic layered structure (2D). CNPs possess unreacted residues of amino groups (hydrophilic groups), mainly along the edges of their 2D structure. As shown in Figure 4d, the unreacted amino groups on the growth side of the CNP film are hydrogen-bonded along the film plane and allow its curled shape to be locked (right). When water is adsorbed on the film surface, those hydrogen bonds are reorganized by the incorporation of water molecules, so that the film is released for straightening (left). The actuation was extremely rapid (50 ms for one curl) and can be repeated >10,000 times without deterioration.

Recently, researchers have discovered that hydrophilic homogeneous MXene has a humidity-driven capability by a moisture gradient similar to that of graphene oxide (Figure 4e) [88]. The maximum bending angle can be as high as 155° at the relative humidity difference of 65%. The humidity-driven and large deformation of the MXene film were formed in situ by the asymmetric expansion of the bilayer structure. Chen et al. [89] prepared a composite-responsive film consisting of relatively hydrophobic RGO and hydrophilic polydopamine (PDA) (Figure 4f). During the trigger of the RGO–PDA film by water with a gradient from one side, humidity-responsive hydrophilic PDA can absorb water on the surface layers of the RGO–PDA film and act as soft “muscle” to activate the swelling locomotion. The rigid RGO sheets, with a hydrophobic nature, hinder the diffusion of water across the film and form the actuator’s “skeleton”, supporting the PDA “muscle” to convert water gradient energy into mechanical motion. The responsive speed of the uniform RGO–PDA thin film triggered by the vapor is very fast, with a bending speed of over 1000°/s, which is significantly faster than the speed of the previous bilayer graphene-based actuator [32].

### 2.4. Crystalline Materials

**Figure 5:**
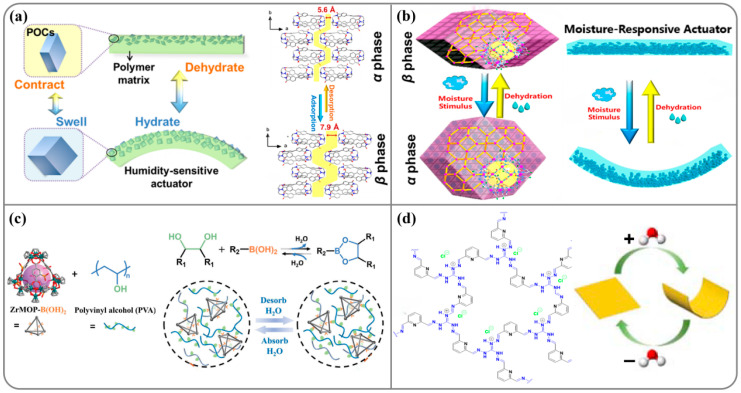
(**a**) Structure and humidity responsiveness of POCs. (**b**) Structure and humidity responsiveness of MOF. (**c**) Illustration of constructing dynamic humidity-responsive HCMOP membranes via covalently crosslinking boric acid-modified Zr-MOP with PVA. (**d**) The chemical structure and the mechanical motion in response to humidity of the self-standing cationic covalent organic framework (TG-DFPCOF) film.

Crystalline materials can also have superior tunable water affinities through the introduction of different types of hydrophilic groups [90,91]. Zhang et al. [92] successfully fabricated flexible porous organic cages (POCs) that can undergo a reversible structural transformation between the *α* and *β* phases upon moisture stimulation (Figure 5a). In summary, the actuator with a filler size of ~0.8 μm, a loading capacity of 45%, and a membrane thickness of ~34 μm showed the best actuating performance. This actuator could perform repeated deformations for at least 20 cycles without any decay. They also found that the metal–organic frameworks (MOFs) can undergo reversible phase transitions in different humidity environments, resulting in a large change in unit cell volume (up to 16.2%) upon hydration (*α* phase) and dehydration (*β* phase) (Figure 5b) [18]. This fast adsorption–desorption process allowed for the harvesting of an extrapolated 7.2 L water per kilogram of MOF per day. A moisture-responsive actuator was further fabricated by hybridizing a polyvinyl alcohol (PVA) polymer with MOF particles. The gradient-distributed structure was beneficial to convert the humidity-triggered expansion/contraction of MOF particles into large macroscopic deformations. The hybrid membrane showed a bending deformation (~0.15 cm^−1^ curvature within 10 min) upon a moisture stimulus. In addition, the cycling test of the actuator under RH = 0–30% suggested that the hybrid membrane can perform repeated deformations (i.e., bending–straightening) for at least 10 cycles without significant decay. Subsequently, they prepared metal–organic polyhedras (MOPs) which exhibited excellent humidity responsiveness (Figure 5c) [17]. The moisture-responsive membranes were facilely fabricated by covalent crosslinking of boronic acid-modified Zr-based MOPs with PVA. In these membranes, MOPs serve as high-connectivity nodes and provide dynamic borate bonds with PVA in hyper-crosslinked networks, which can be broken/formed reversibly upon the stimulus of water vapor. An increase in the Zr-based MOP load significantly increases the humidity sensitivity, reflected in the faster bending response and recovery speed. The hybrid membrane could achieve a bending angle of *θ* = 178° in as short as 10 s after exposure to water vapor and could rapidly recover to the *θ* = 22° position once removed within 18 s.

Afterwards, Trabolsi et al. [33] prepared a self-standing cationic covalent organic framework (TG-DFPCOF) film (Figure 5d). Because hydrogen bonding and ionic surface coverage are present throughout the COF network, this material facilitates the rapid adsorption and desorption of water vapor, leading to an ultrafast actuating response rate of less than 1 s. Upon approaching the top surface of the cup, the film curled up in response to the high humidity, bending significantly up to an angle of *θ* = 178° within 0.22 s and then returning to its original shape once removed from the cup. The complete folding and unfolding process of the TG-DFPCOF actuator occurred in only 0.69 s. Fast water adsorption and transfer are the main advantages of crystalline material sorbents, which provide an efficient water adsorption site supply and diffusion channel. However, processing crystalline materials within hierarchical structures is of high importance in translating the order on the molecular level to the macroscopic scale. Research on crystalline materials with the rational design of a water transport channel has been extensively studied in recent years, which provides meaningful insight into the development of an energy harvesting system [93,94,95].

## 3. Practicality and Application

By harnessing the chemical potential energy inherent in the diffusion process of atmospheric humidity and converting it into readily available electrical energy, a novel source of potential energy is being unveiled, boasting promising applications within the realm of renewable energy [96,97,98,99,100]. Based on the fact that most moisture-sorption materials have biocompatibility, flexibility, excellent mechanical, self-continuity, and some other outstanding properties, the moisture-sorption actuators made from those materials demonstrated vast applications in intelligent wearables, biosensing technologies, smart switching, and so on.

**Figure 6:**
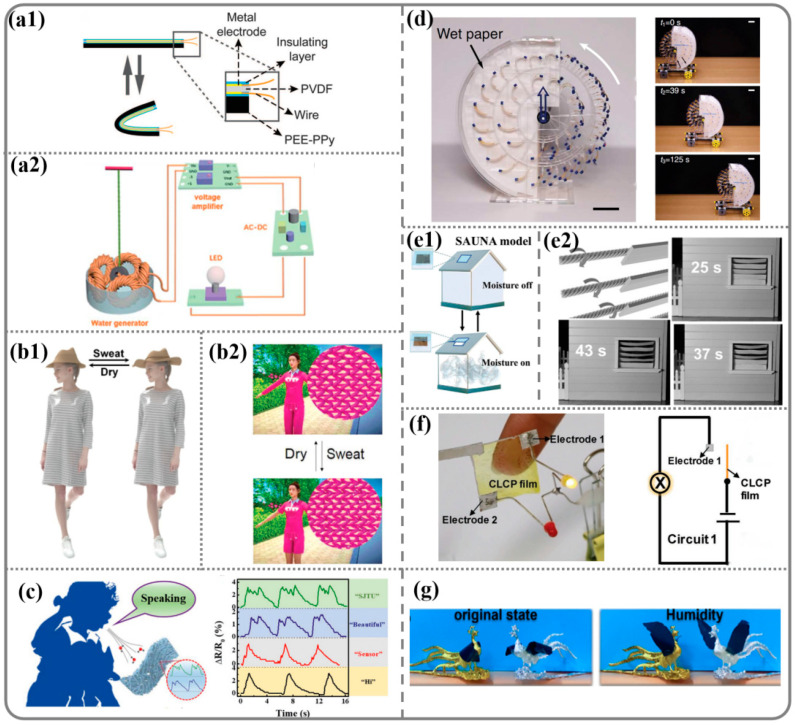
(**a**) Design and performance of a humidity-driven generator. (**a1**) Sketch of the film-based generator. (**a2**) Schematic diagram of the fiber-based generator. (**b**) Intelligent wearables. (**b1**) Model of moisture-managing hat responding to moisture changes after long duration in hot weather resulting in forehead perspiration. (**b2**) Sequential photos showing that smart clothing can change macro-shape or micro-structure to achieve moisture and thermal management. (**c**) Schematic illustration and response signals of the multilayer graphene/polyamide (MG/PA66) humidity sensor (MPHS) for human exhaled air detection during speaking. (**d**) A miniature car driven by a rotary engine. (**e1**) Moisture-responsive performance of a smart curtain. (**e2**) Smart window and louver in response to moisture. (**f**) A sensor which was assembled with conductive materials and LED lights. (**g**) Optical images of the phoenix’s wings flapping and completing the opera under the stimulation of moisture.

Energy harvesting is one of the important applications of humidity-responsive materials. The material absorbs moisture to produce mechanical energy, which is subsequently transformed into electrical energy for the purpose of energy harvesting. This generation device primarily encompasses piezoelectric transducer nanogenerators [8,20,21,25] and an electromagnetic generator for achieving magnetoelectricity [1,2,56,57]. Langer’s research team [20] assembled PEE–PPy actuator membranes with the piezoelectric material PVDF (Polyvinylidene difluoride) to form a generator (Figure 6(a1)). Within 7 min of charging, the voltage of the capacitor was saturated to ~0.66 V. The electrical energy can be stored in a capacitor to provide power for micro- and nano-electronic devices. Wang et al. [57] fabricated a fiber-based water generator, which generated electricity under water stimulation. The schematic diagram of the fiber-based generator is shown in Figure 6(a2). The LED did not light up as the twisted fiber was stationary. However, when the fiber was rotated at a high speed, the LED was brightly lit, and the output DC was as high as 2.8 V. 

Zhang et al. [51] fabricated an origami pristine silk fibroin (SF) film. In a high-humidity environment, water molecules are absorbed from the bottom of the SF film. With the additional absorption of water, the increased stress induces SF origami to move along axis-x and axis-y. Eventually, the SF origami undergoes a complete flip and jumps. Furthermore, this film can be used as a hat lining to manage perspiration on the forehead while staying fashionable (Figure 6(b1)). Liu et al. [101] reported a textile from these silk fibers. As shown in Figure 6(b2), the sleeves of the smart textile shrink in the warp (vertical) direction when humidity increases (for example, due to perspiration or humid environment), and then expands when humidity decreases. The investigated smart sleeves generated a large contraction (45%) when exposed to moisture or sweat, and then recovered to its initial length when the environment became dry. This moisture-responsive textile, which can change its macro-shape or micro-structure, promises to be very effective in achieving such functions for moisture and thermal management for increased comfort between the skin and fabric.

The human body’s skin plays a role in the overall metabolism and contributes to 18–20% of the total water content, enabling sweat on the skin surface to function as a remote noncontact control source for a humidity sensor. Yang et al. [29] reported a flexible high-sensitivity humidity sensor, fabricated by anchoring multilayer graphene (MG) into electro-spun polyamide (PA) 66 (MPHS). Figure 6c shows a schematic illustration of the MPHS for human exhaled air detection during speaking. Various repeated responses of the MPHS to words with different numbers of syllables were recorded. Each syllable had a corresponding characteristic peak with different intensities in the recording signals when the subject spoke certain words.

For mobile devices and wearable technology, it is essential to develop lightweight and compact power sources that do not rely on traditional charging methods. Goodnight et al. [56] reported a steam-driven actuator that could propel a small car (weighing 0.1 kg) forward as the water in the car evaporated (Figure 6d). Wu et al. [102] reported a hydrophobic/hydrophilic linear polymer formed with a semi-intercross crosslinking network. Such a copolymer exhibits excellent humidity-sensitive, mechanical properties and water resistance. Based on these characteristics, windows that open automatically are successfully developed (Figure 6(e1)). Peng et al. [85] focused on investigating the mechanical behavior of carbon nanotube fibers under the influence of water and humidity (Figure 6(e2)). They constructed multi-level helical carbon nanotubes to create numerous nanoscale and micron-scale pipe structures within the fibers, enabling an efficient and rapid solvent penetration into the fiber interior. This resulted in the swift, large-scale reversible contraction and rotation of the fibers. The fibers achieved a responsive contraction and rotation to water, leading to the development of smart curtains capable of detecting changes in humidity. 

In daily life, the storage of different items has strict requirements for air humidity, and it is difficult to perceive tiny changes in humidity. By converting humidity signals into visually perceptible signals, people can more intuitively detect humidity changes. This signal conversion can be achieved through intelligent actuation mechanisms. Yu et al. [75] developed a hydrophobic polymer actuator that responds to humidity and exhibits crosslinked liquid crystal properties. When this thin film is connected to a circuit, the variations in humidity close the circuit and activate an LED light, thereby creating an alarm effect (Figure 6f). 

Moreover, there are many humidity-responsive materials in nature. For example, pinecones open and close in different humidity environments. At present, humidity-responsive materials have also demonstrated a unique performance in imitating biological behaviors. For instance, Dai et al. [103] applied the humidity-responsive film as an actuator in the Chinese shadow puppet show “Feng Qiu Huang”. The wings of the phoenix are initially closed; then, when moisture is introduced, the humidity-responsive film immediately bends upward, resulting in open phoenix wings (Figure 6g). The applications demonstrate the outstanding performance and enormous potential of flexible actuators, opening new avenues for promoting traditional culture.

## 4. Outlooks and Challenges

In this review, we summarize the recent progress in moisture-sorption materials. In general, moisture-responsive actuators are mainly composed of thin films and fibers [2,85,104,105]. With the involvement of more researchers from various interdisciplinary fields, the types of moisture-sorption materials have become increasingly diverse. For large-scale applications of moisture-responsive actuators, it is necessary for the moisture-sorption materials to possess high mechanical strength, sensitivity, and controllable moisture absorption level. Additionally, the development of simple fabrication methods is also crucial. Based on a review of the recent literature, the characteristics and future directions of moisture-sorption materials can be summarized as follows:

(1) Biomaterials: In nature, an abundance of biomass raw materials with renewable, biocompatible, biodegradable, and excellent moisture absorption properties exists. However, the complex secondary processing required for biomass raw materials often limits their large-scale production. Therefore, there is a need to improve the mechanical properties and long-term stability and reliability of the prepared actuators.
(2) Synthetic polymers: For synthetic polymers, the number, type, and positioning of hydrophilic groups can be tailored to achieve controllable water absorption and water adsorption/desorption capabilities. The modifiable flexibility and rigidity of molecular chains, as well as crystallinity and orientation, confer excellent mechanical properties and versatility for diverse applications. Furthermore, through compositing with other materials (CNT, COF, MOF, MXene, GO, and so on), the polymer can acquire unique properties and will be ideal for humidity-responsive actuators. Nevertheless, the current range of humidity-absorbing polymers is limited, with the narrow scope of humidity absorption being a primary constraint on its advancement. Therefore, the development of innovative humidity-absorbing polymers holds great significance for harnessing green energy.
(3) Nanomaterials: The materials are primarily categorized as 1D and 2D nanomaterials, exhibiting either hydrophilic or hydrophobic properties. In the case of hydrophobic nanomaterials, surface modification with hydrophilic properties is necessary prior to layer-by-layer stacking for the formation of a moisture-responsive actuator. Nevertheless, these membrane actuators often exhibit low durability and are susceptible to delamination. Hence, enhancing the longevity of these materials remains an imminent challenge.
(4) Crystalline materials: Crystalline materials have superior tunable water affinities. And the hydrophilic groups can be uniformly installed in the skeleton of the crystal materials to form ordered molecular arrays, making energy transfer between moisture-stimulus signals and responsive sites much faster and more efficient. However, most of the current crystalline materials are in the form of particles or powders, which must be blended with other polymers to form a humidity-responsive actuator. Although physically blending is conveniently used, it suffers from deficiencies from aggregation and precipitation, resulting in poor performance. As a result, improving the dispersion of crystal materials in polymers is a key factor in the future development of moisture-absorbing materials.
